# Glioma stem cells and their roles within the hypoxic tumor microenvironment

**DOI:** 10.7150/thno.41692

**Published:** 2021-01-01

**Authors:** Nathaniel H. Boyd, Anh Nhat Tran, Joshua D. Bernstock, Tina Etminan, Amber B. Jones, G. Yancey Gillespie, Gregory K. Friedman, Anita B. Hjelmeland

**Affiliations:** 1 Department of Cell, Developmental, and Integrative Biology, University of Alabama at Birmingham, Birmingham, AL.; 2 Department of Neurosurgery, Northwestern University, Chicago, IL.; 3 Department of Neurosurgery, Brigham and Women's Hospital, Harvard Medical School, Boston, MA, USA.; 4 Department of Chemistry, University of Alabama at Birmingham, Birmingham, AL.; 5 Department of Neurosurgery, University of Alabama at Birmingham, Birmingham, AL.; 6 Division of Pediatric Hematology and Oncology, Department of Pediatrics, University of Alabama at Birmingham, Birmingham, AL.

**Keywords:** acidic stress, glioma, hypoxia, cancer stem cells, tumor microenvironment

## Abstract

Tumor microenvironments are the result of cellular alterations in cancer that support unrestricted growth and proliferation and result in further modifications in cell behavior, which are critical for tumor progression. Angiogenesis and therapeutic resistance are known to be modulated by hypoxia and other tumor microenvironments, such as acidic stress, both of which are core features of the glioblastoma microenvironment. Hypoxia has also been shown to promote a stem-like state in both non-neoplastic and tumor cells. In glial tumors, glioma stem cells (GSCs) are central in tumor growth, angiogenesis, and therapeutic resistance, and further investigation of the interplay between tumor microenvironments and GSCs is critical to the search for better treatment options for glioblastoma. Accordingly, we summarize the impact of hypoxia and acidic stress on GSC signaling and biologic phenotypes, and potential methods to inhibit these pathways.

## Introduction

Glioblastoma (GBM), also known as a World Health Organization grade IV astrocytoma, is the most common and aggressive primary brain tumor in adults. From 2012-2016, the average incidence of malignant brain tumors in the U.S. was 7.08 per 100,000, and GBM accounted for about 15% of all central nervous system tumors, and close to half of all malignant brain tumors diagnosed. GBMs have a disproportionate incidence rate by sex and race, occurring 1.58 times more often in males than females, 1.95 times more often in whites than blacks, and 2.39 times more often in whites than Asian or Pacific Islanders 1. Although past studies have significantly increased our understanding of the signaling pathways and molecular processes involved in gliomagenesis, the prognosis remains dismal despite a multimodal approach utilizing maximal surgical resection, adjuvant radiation and chemotherapy using the DNA alkylating agent, temozolomide (TMZ) 2. Patients receiving standard of care have a median survival of 14.6 months, with only around 10% of patients surviving 5 years or longer 3. The failure of current treatments in substantially prolonging life expectancy highlights the importance of increasing our understanding of the pathobiology promoting/driving GBM growth, recurrence and therapeutic resistance.

## Glioblastoma intratumoral heterogeneity

A characteristic that tumor cells share with non-neoplastic stem cells is their ability to proliferate indefinitely. Accordingly, many tumors appear to be maintained via a hierarchical organization that consists of slowly-dividing stem cells, precursor cells, and differentiated cells 4, 5. In line with such evidence, only a subset of cancer cells has the ability to form tumors within immunodeficient mouse models when derived from multiple cancers 6. In brain tumors, this subset of cells, known glioma stem cells (GSCs), exhibit self-renewal that can be measured *in vitro* via neurosphere formation assays and the expression of molecular markers (e.g. SOX2, NANOG, CD15, CD133) in symmetric and asymmetric division studies 7. Indeed, GBMs have long been known to express both neural and glial markers, which suggest a cell subset with neural stem cell-like characteristics 4, 8. While a number of methods have been used to isolate GSCs, fluorescence-activated cell sorting (FACS) using the cell surface marker CD133 remains one of the most characterized 9-11. Isolation of CD133 positive and negative populations in GSCs first revealed differences in tumor propagation in xenograft mouse models 9, 12. Subsequent investigation revealed co-expression of CD133 with Nestin and other canonical neural stem cell markers, and increases of CD133 expressing GSCs post-irradiation, indicative of a therapy-resistant, stem-like fraction 13. In addition to self-renewal and a multilineage differentiation capacity, GSCs exhibit invasive and angiogenic potential, as well as therapeutic resistance, which will be discussed in more detail.

Through genetic and epigenetic characterization, a number of adult GBM subtypes have been defined 14. Of note, single cell RNA-sequencing has clearly determined that multiple subtypes exist within one GBM tumor 4, 15, suggesting that the vast heterogeneity within these tumors may complicate the ultimate goal of preventing recurrence of GBMs. Heterogeneity is further complicated by the presence of GSCs that are capable of propagating tumors in immunocompromised mice, as well as maintaining the expression of neural stem cell markers and/or dividing asymmetrically to generate more differentiated progeny 9-11. Critically, GSCs resist radiation- and chemotherapy-induced cell death to a greater extent than the bulk tumor, with data from mouse models indicating that a quiescent GSC fraction is directly associated with therapeutic resistance and tumor recurrence. These GSCs reside in multiple niches that include those located in tumor microenvironments (e.g. low oxygen tension [hypoxia], acidic stress, and/or nutrient restriction) that promote the characteristics mentioned above and contribute to intratumoral heterogeneity, which leads to major challenges in treatment 16. Thus, understanding GSC molecular signaling pathways in the context of the tumor microenvironment is of paramount importance for developing novel treatment paradigms for this intractable central nervous system neoplasm 17, 18.

GSCs assist in establishing the tumor microenvironment through complex crosstalk within their niche. The two most commonly described niches in which GSCs have been characterized are the perivascular and the perinecrotic niches 19-21. Both of these niches deliver instructive cues that serve to maintain GSCs and stimulate cellular plasticity towards a stem-like phenotype 22, 23. Perinecrotic niches are enriched for cells expressing molecular markers of both hypoxia and GSCs (e.g. SOX2, NANOG, CD133) 24, 25, suggesting a connection between the tumor microenvironment and differentiation state of cells. Similarities between GSC-regulated and hypoxia-induced biology strengthen these apparent connections. For example, angiogenesis and invasion are well-established pro-tumorigenic cellular behaviors induced by hypoxia, while tumors that arise from GSCs are highly vascular and more invasive as compared to tumors generated from non-GSCs. As GSCs are responsible for tumor propagation and invasion, promote angiogenesis, are resistant to therapy, and contribute to tumor recurrence, it is essential to develop therapeutic agents capable of targeting GSCs.

Other GSC-associated niches, the peri-hypoxic, peri-immune, and extracellular matrix niche are reviewed in-depth in Aderetti et al. 26. Briefly, the peri-hypoxic niche promotes the stemness capability of GSCs, as well as promotes the acidification of the tumor microenvironment, which stabilizes HIF 27. Acidosis can also be induced through elevated carbonic anhydrase, lactate, and ion transporter activity 28. The peri-immune niche is maintained through upregulated activity of tumor associated macrophages with enhanced immunosuppressive activity 29, and the ECM niche has immense interaction with the other distinct niches, and its components can influence GSCs ability to synthesize their own ECM in both a direct and indirect manner 30. These physiologic niches comprise an integrated network that can promote overall GSC maintenance, and each component will be described in-depth in the current review.

## Hypoxia and acidic stress as important factors in the brain tumor microenvironment

**Figure 1:**
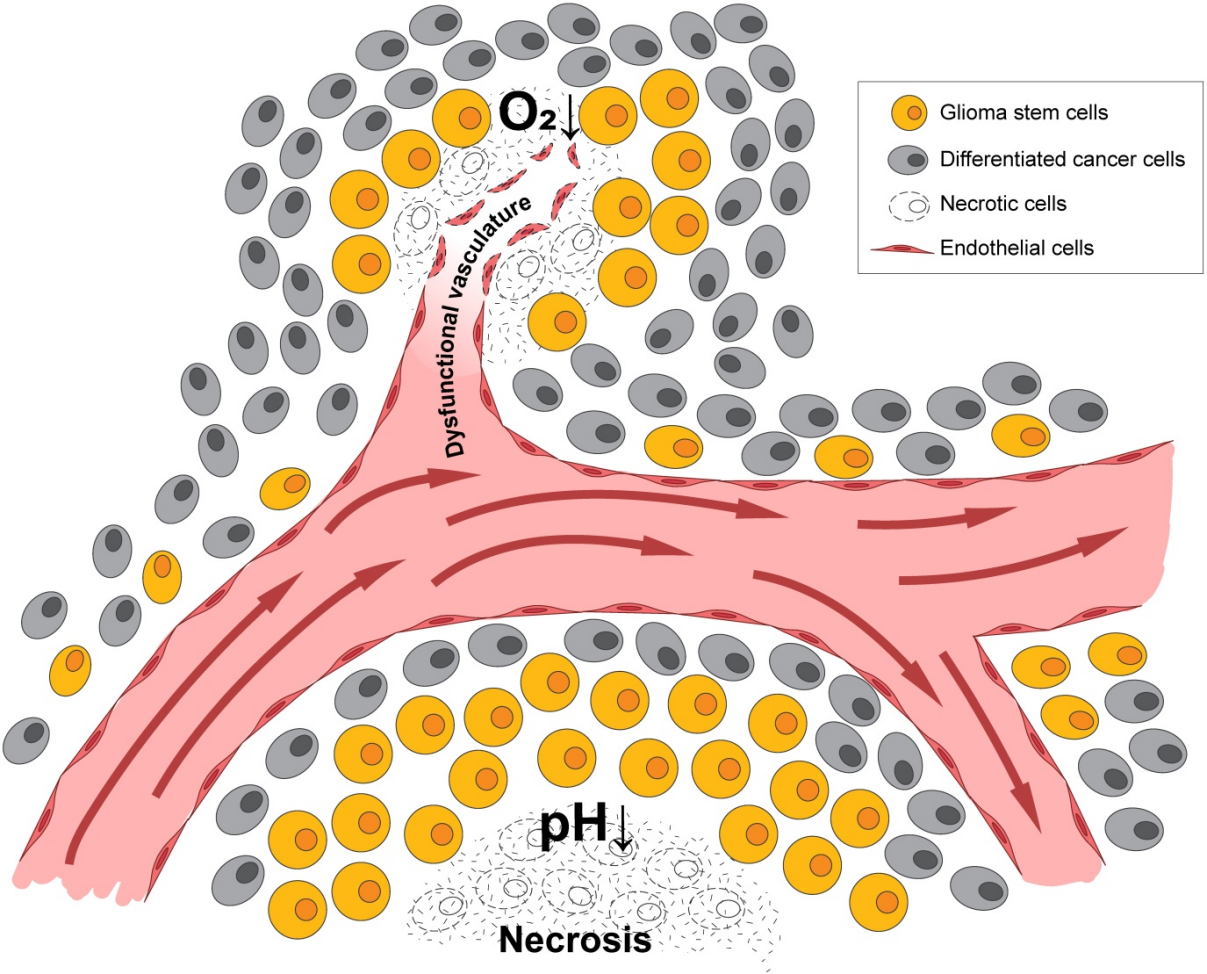
** Hypoxia and acidic stress exist in microenvironmental niches for brain tumor initiating cells.** Normally represented together as necrotic zones, they are also found separately in brain tumors and can independently affect biologies and gene expression patterns.

A pathologic hallmark of GBMs, pseudopalisading necrosis, often occurs near a collapsed blood vessel, and tends to be surrounded by cells surviving in a hypoxic and often, acidic zone 31. In patients with GBM and in murine xenograft models of GBMs, it has been revealed that a median partial pressure of oxygen (pO_2_) of 5-9 mm Hg and an acidic pH of 6.8 or lower 32-34 often existed within these tumors. In contrast, oxygen tension in arterial blood is approximately 70-100 mm Hg, while the normal brain often measures 25-40 mm Hg with some niches being even lower. Thus, there is a significant difference between physiologic oxygen tension and pH, and that of the tumor. While hypoxia and low pH often occur simultaneously, studies in xenograft models using microscopy with phosphorescence quenching to monitor pO_2_, and ratio imaging to measure pH, have demonstrated these microenvironments occur independently 35, as illustrated in **Figure 1**. As hypoxia and/or low pH correlate with many aspects of tumorigenicity, including therapeutic resistance, patient survival, and tumor invasion, understanding tumor microenvironmental effects on GBM growth and recurrence is critical 36.

The presence of hypoxia promotes the use of anaerobic glycolysis to generate energy and essential precursors (nucleic and amino acids as well as lipids) required for cell growth 37. Anaerobic glycolysis results in the production of acidic metabolites, including lactic acid, and facilitates an altered pH balance in solid tumors wherein extracellular pH is lower than intracellular pH. There is a marked reduction in median solid tumor partial pressure of oxygen (pO_2_) and extracellular pH compared to non-neoplastic tissue, as well as the development of regions of extreme hypoxia and low pH 38. However, oxygen tensions vary significantly depending on the non-neoplastic tissue, indicating the importance of establishing a physiologic normoxia control when designing experiments to uncover differences in these microenvironmental conditions. While there are some innovative studies attempting to address this problem, the overwhelming majority of *in vitro* experiments continue to be performed in atmospheric oxygen (~21% O_2_ or 159 mmHg) using buffered media to minimize pH changes (typically pH 7.4) 39. Recognizing the limitations of these *in vitro* approaches, a shift towards improved modeling of physiologic microenvironments is desperately needed.

Hypoxia-responsive signaling in both tumors and nonmalignant tissue is mediated through transcription factors called hypoxia-inducible factors (HIFs). HIFs exist as heterodimers, consisting of an alpha and a beta subunit. The beta subunit is constitutively expressed in all cells, while the alpha subunit exists in three isoforms, which are unstable and rapidly degraded in the presence of oxygen 40, 41. Under well-oxygenated conditions, prolyl hydroxylases (e.g. PHD1, 2, and 3) hydroxylate proline residues on HIFα subunits. The hydroxylation site acts as a substrate for Von Hippel Lindau factor (VHL), which ubiquitinates the HIFα subunit and targets it for proteasomal degradation. However, under hypoxic conditions, the interaction between VHL and the HIF alpha subunit is disrupted. Free iron and α-ketoglutarate are required for the hydroxylation of the proline residues. In hypoxia, free iron is chelated, and the alpha subunit cannot be hydroxylated and is therefore stabilized 42, 43. The HIFα subunit can then translocate to the nucleus, bind to the beta subunit, and subsequently recognize hypoxia-responsive elements (HREs), which are consensus sequences (5'-ACGTG-3') in the promoter region of identified target genes. The binding of HIFs to these sequences upregulates the transcription of genes controlling cell survival, glycolysis, pH regulation, angiogenesis, migration, and invasion 40, 44. This becomes particularly important in secondary GBMs, as many possess a mutation in the Isocitrate Dehydrogenase (IDH) enzyme that is responsible for the conversion of isocitrate to α-ketoglutarate 45. IDH mutations disrupt enzyme functionality, causing the production of D-2-hydroxyglutarate, a competitive inhibitor of α-ketoglutarate. Through this mechanism, HIF levels may be increased 46 and the mutation is associated with global methylation changes 47. Furthermore, hypoxia or low pH in normoxia in glioma cells increased levels of L-2-hydroxyglutarate (a mirror-image enantiomer) and acidic stress increased HIF stabilization 48, 49.

Two well-characterized HIF alpha isoforms are HIF1α and HIF2α. Suggesting that these two isoforms program distinct responses to changes in the microenvironment, HIF2α expression can be stabilized at higher oxygen levels (approximately 5%) than the more severely hypoxic conditions (less than or equal to 1%), where HIF1α is induced 50-52. These two isoforms both bind to HRE sequences but can have distinct target genes. For example, genes preferentially induced by HIF2α have ETS binding elements adjacent to HREs 53. HIF2α can also stabilize MYC and myc associated protein (MAX) interactions to enhance myc transcription 54, 55. In contrast, HIF1α appears to bind to MAX to prevent MYC signaling. Thus, the isoforms of HIFα can function to activate distinct signaling compartments, although there are many common, HRE containing HIF targets such as Vascular Endothelial Growth Factor (VEGF), a well-known proangiogenic protein.

## Hypoxia and/or HIF signaling promote GSC maintenance

GSCs are identified using cell surface markers or cell selection techniques that take advantage of phenotypes increased in this cellular subset, with sorting or validation using GSC markers that can include CD133, OCT4, and SOX2 among others 7, 9, 12, 13, 52, 56, 57. Exposure to hypoxia upregulates these same canonical stem cell genes and facilitates plasticity towards a stem-like state (as indicated by increased expression of stem cell markers including CD133) 52, 58-64. Furthermore, the hypoxic microenvironment may help GBM cells under certain inhibitor treatments to maintain their stem-like phenotype, while normoxia does not 65. Hypoxia was also shown to promote glycosylation of CD133, which could play a role in the process of anti-hypoxia-mediated apoptosis 66. This is notable as antibodies used to isolate CD133 during flow cytometry are often specific to the glycosylated form of the protein. Other GSC markers that increased in expression under relatively low oxygen levels for GBM include podoplanin, BMI-1, and Nestin 62. However, Sox2 expression increased only in *in vivo*-like multicellular tumor spheroids derived from GBM short-term culture with tumor stem cell properties, indicating that tumor cell phenotypes associated with stemness and chemoresistance may depend on the oxygen tension surrounding that particular tumor cell as well as cellular interactions 62. Thus, hypoxia increases expression of many GSC markers, which may contribute to the growth of the tumor.

**Figure 2:**
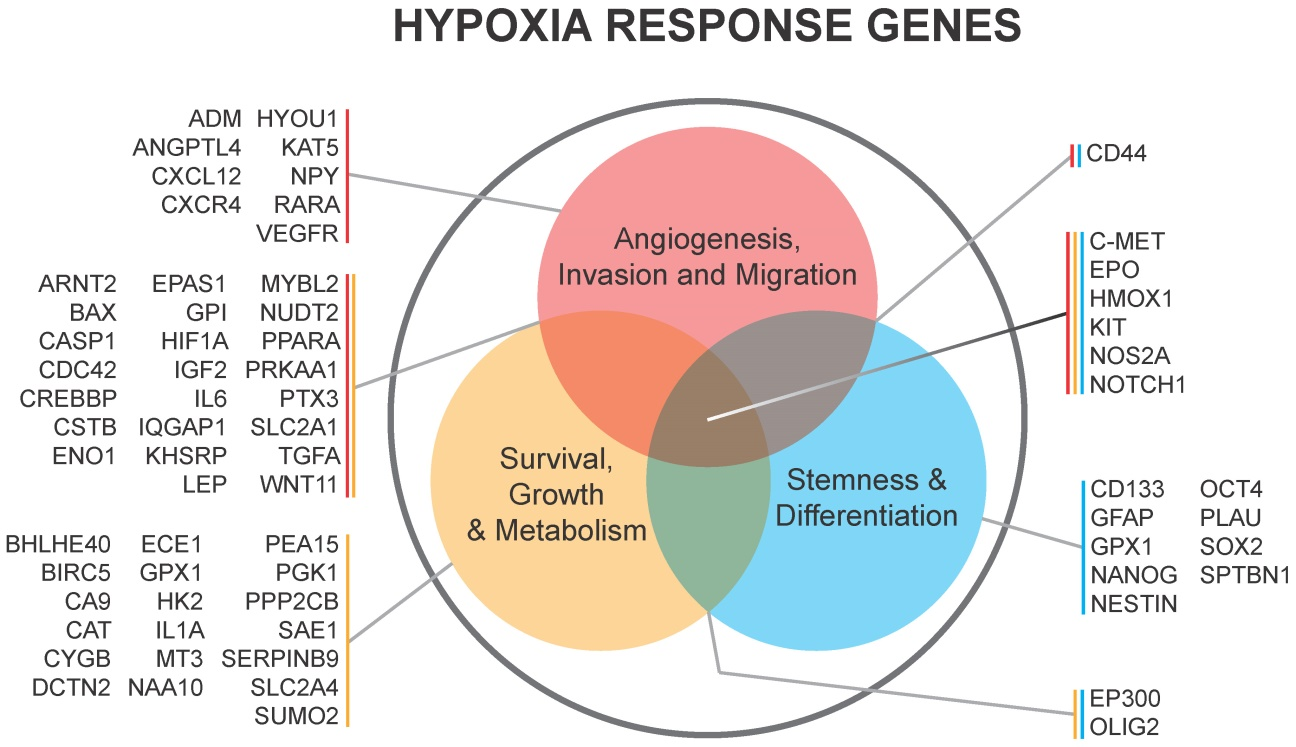
** Hypoxia response genes in glioma and their subsequent downstream biologies relevant to BTICs** in Li et al. 2009 and Keith et al. 2011.

HIF1α and HIF2α are both critical for GSC function and can be expressed in different patterns dependent upon the level of hypoxia as mentioned above. The stabilization of HIF1α leads to the expansion of the GSC population within the bulk of the tumor, which is, in part, mediated by the extracellular signaling related kinase (ERK) and the PI3K/AKT pathways 58. Conversely, RNA interference-mediated silencing of HIF1α depleted the self-renewal capacity of GSCs and led to a reduction of tumorigenic potential *in vivo* 58. HIF1α is also a negative regulator of bone morphogenetic proteins 67, which are known to signal differentiation of GSCs towards an astrocyte lineage 68. HIF1α/STAT3 co-activator complex induces the transcription of Vasorin, which, in turn, stabilizes Notch1 and augments Notch signaling, promoting GSC maintenance 69. Additional evidence supports the importance of HIF1α activation of the JAK1/2-STAT3 transcriptional program, including via VEGF as an important autocrine factor, to enhance GSC maintenance 70. Thus, HIF1α promotes GSC self-renewal and represses differentiation to increase GBM growth. A list of genes regulated by hypoxia in GSCs is shown in **Figure 2.**

Under less extreme hypoxia, when HIF2α is preferentially expressed in GSC populations, HIF2α levels were regulated not only via post-translational modification but also through increased transcription 50. HIF2α activity is repressed by the DEAD box protein DDX28 71, while stability is also regulated by the transcriptional regulator Inhibitor of DNA Binding 2 (ID2) 72. In normoxia, dual specificity tyrosine-phosphorylation-regulated kinases (DYRK1A and DYRK1B) phosphorylated ID2. Phosphorylated ID2 can no longer interact with the VHL ubiquitin ligase complex, so HIF2α is ubiquitinated and degraded. In hypoxia, phosphorylation of DYRKs and ID2 is decreased, resulting in HIF2α expression. HIF2α stabilization upregulates some stem cell factors, including Oct4, Sox2, and Nanog, while knockdown of HIF2α reduced the self-renewal capacity of GSCs *in vitro* and decreased tumor growth *in vivo* 50, 58, 59, 63, 73-75. Recently, CD44 was shown to be cleaved into an intracellular domain that interacts with HIF2α and promotes HIF dependent hypoxic signaling 76. As CD44 is associated with mesenchymal GSCs, this data highlights the potential for heterogeneous hypoxic responses in GBM. Together, the data indicate that hypoxia and HIFs play important roles in maintaining the GSC phenotype through multiple mechanisms 50.

## The relationship between acidic stress and GSCs

Although low pH is recognized to be an important component of the tumor microenvironment and has been shown to exist in the absence of hypoxia *in vivo*, relatively few studies focus on the biological effects of acidic stress in GBM or other solid tumor cells. The extracellular pH of solid tumors including GBMs has been measured as low as 5.9 with an average pH of 6.8, whereas the normal brain pH is approximately pH 7.1 77. Considering the protumorigenic properties of tumor acidification and the altered pH gradient in cancer (pH_Extracellular_< pH_Intracellular_), drugs altering proton export or production, and buffer therapies are being explored as novel treatments 78-86, as well as metabolic enzymes that contribute to the production of lactic acid as a result of a glycolytic shift 87. Sodium bicarbonate buffer therapies have decreased tumor growth and metastasis in breast and prostate cancers 81, 85, 88, 89, and carbonic anhydrase IX inhibition effectively targeted breast cancer initiating cells *in vivo* and improved the efficacy of immunotherapy 90, 91. Thus, there are multiple strategies that could be employed to modulate pH with only a limited number having been explored in GBM, particularly in the context of standard of care.

Applying acidic stress to GBM cells upregulates VEGFA mRNA via the ERK1/2 and MAPK pathway, which enhances AP-1 binding to the VEGF promoter 92. Importantly, VEGF can be upregulated by hypoxia or acidic stress independently, including in GSCs 11, 35, 93. These data suggest that both regions are involved in GBM neovascularization and could be targeted for anti-angiogenic therapies. The VEGF antibody bevacizumab is already approved for treatment of GBM 92, 94-97, but bevacizumab is ineffective as a standalone therapy for newly diagnosed GBM patients 98, 99. However, studies have yet to assess the efficacy of combinatorial treatments of bevacizumab with tumor pH gradient-targeting drugs.

Acidic stress also promotes glioma stem cell phenotypes independently of hypoxia, but these may still involve HIF2α expression. Exposure of GBM cells to acidic stress increased GSC marker expression as well as self-renewal and tumor growth 93, 100. Acidosis also functions in concert with hypoxia to upregulate HIFs in GSCs through an alternative PHD/VHL-independent pathway involving the stress-induced HSP90 chaperone protein 27. Importantly, acidic stress is also likely to shape the epigenetic response within cellular programs, as it has been shown that acidic stress represses the epigenetic reader chromodomain helicase DNA binding domain protein 7 (CHD7) in GSCs 101.

CAIX and CAXII contribute to cellular growth by maintaining extracellular acidification and an alkaline intracellular pH in response to tumor acidosis 102. CAIX is upregulated in glioma, and acidosis leads to an increase in CAIX in GBM cells involving HIF transcriptional machinery that is independent of hypoxia 103, 104. CAIX protein expression is an independent poor prognostic factor in GBM patients 105, suggesting the importance of understanding its function and the potential of successfully targeting the pH regulator. After CAIX knockdown, cell attachment, migration, and chemotherapeutic resistance are reduced, while apoptosis increases 106. CAIX si/shRNA inhibits GBM growth and enhances anti-VEGF therapy, while the CAIX/XII small molecule inhibitor SLC-0111 in combination with TMZ prevents GSC enrichment and increases survival 82, 107, 108. CAXII inhibitors also reduced the growth of TMZ-resistant GSCs by inhibiting P-glycoprotein-mediated drug efflux 109. TMZ resistance in GSCs was further shown to be CAII mediated, with the broader CA inhibitor acetazolamide showing efficacy in combination with TMZ in xenograft models 110, 111. Furthermore, CAIX is suggested as a target in GBM for CAR T-cells that could be effective against GSCs 112. A recent modeling study also suggested modulating pH to increase glioma TMZ sensitivity 113. Importantly, regions of acidic stress can cause GBM tumor cells to exhibit differential growth patterns depending on their genetics, as well as differential responses to chemotherapeutics 114. For example, induction of WAF1 by acidosis, which results in cell cycle arrest, does not occur in p53 mutated cells and represents a mechanism by which acidosis could select for certain advantageous mutations in GBMs 115. GBM cells with loss of p53 also had reduced responses to the CAIX/XII inhibitor SLC-0111 107. These reports suggest the roles for acidic stress and carbonic anhydrases in clonal selection and therapeutic resistance.

**Figure 3:**
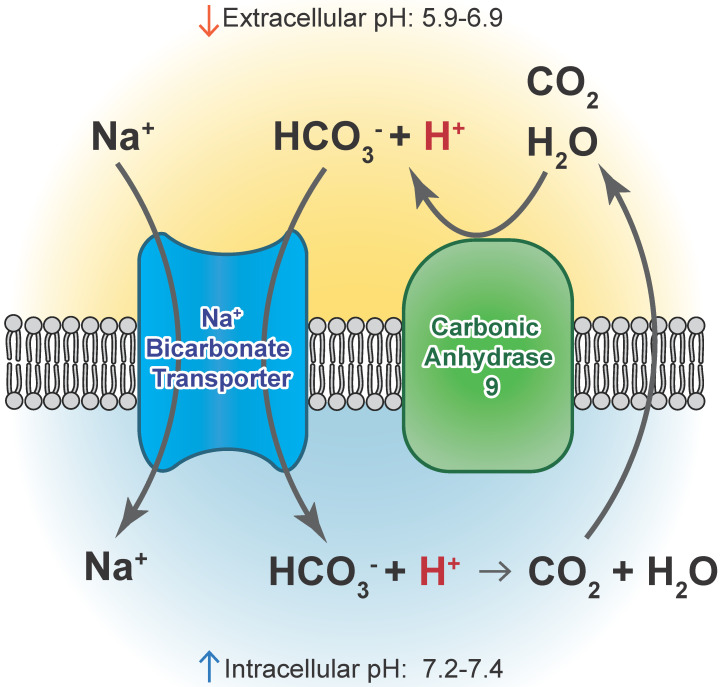
** Carbonic anhydrase 9 functions to modulate extracellular and intracellular pH** by generating protons and bicarbonate via hydrolysis of carbon dioxide and water. This enzyme works in tandem with sodium bicarbonate transporters that import bicarbonate into the cell to buffer intracellular pH.

Acidic stress-induced changes in metabolism occur in GBM, and GSCs can adapt to different tumor microenvironments via shifts between glycolysis and oxidative respiration 116-118. GBM cells adapted to low pH exhibited increased activation of AMPK, leading to a higher rate of glycolysis and inhibited oxygen consumption, indicative of the common glycolytic phenotype in brain tumor cells 119. Conversely, Hu et al. observed increased respiration under acidic conditions that was driven by an increase in CYP24A1 expression. CYP2A1 metabolizes the active form of Vitamin D, an inhibitor of GSC phenotypes in these experiments. The authors did not report measurements of extracellular acidification rate that would describe glycolytic metabolism, which could explain the discrepancy. The authors also observed a co-localization of CYP24A1 with CAIX *in vivo*, suggesting that this enzyme is active in the necrotic zones of GBM tumors 100. In our own experiments, treatment with the CAIX inhibitor SLC-0111 decreased tumor growth in association with a reduced metabolic state 107. An overview of CAIX function is illustrated in **Figure 3.**

## Hypoxia-induced angiogenesis and GSCs

One of the most well-defined biological effects of hypoxia and HIFs in solid tumors including GBMs is the promotion of new blood vessel formation, or angiogenesis. While gliomas first support their growth by taking advantage of existing brain blood vessels in a process called vascular co-option, hypoxia will still ultimately occur as the tumor grows. A hypoxic microenvironment has also been linked to defective neovasculature, resulting in significantly poorer prognosis 120. GSC-derived tumors have increased vessel density and blood perfusion in comparison to tumors that form from non-GSCs 50, 121, 122, suggesting differences in angiogenesis. Although controversial, hypoxia, including that induced by chemotherapy, may promote the transdifferentiation of GSCs to endothelial cells, creating tumor-derived blood vessels (see additional details below) 123-126. The increase in angiogenic properties of GSCs is, at least to a certain degree, due to the upregulation of VEGF in GSCs in comparison to non-GSCs isolated from the same tumor 11, 50, 121, 122. VEGF protein production is regulated by both HIF1α and HIF2α in GSCs, whereas only HIF1α impacts VEGF levels in non-GSCs 50. Furthermore, VEGF production is regulated by OCT4 that is expressed in GSCs through the AKT-HIF1 pathway, where AKT is an oncogenic signaling factor and HIF1 is a transcription factor that is upregulated under hypoxia 127. VEGF produced by GSCs can be secreted in extracellular vesicles called exosomes 128, suggesting a mechanism for more distant VEGF delivery that may regulate tumor angiogenesis and blood-brain barrier permeability 129. Indeed, VEGF has been shown to be induced via delivery of miR-21 in GSC-derived exosomes 130.

GSCs express VEGF receptor 2 (VEGFR2), permitting an autocrine loop of VEGF/VEGFR signaling that can become further activated with increased VEGF under hypoxia 131. VEGF/VEGFR2 signaling may, in turn, support the self-renewal of GSCs: VEGF was shown to activate STAT3 and subsequently induce MYC and Sox2 expression 132. GSCs transdifferentiation towards an endothelial or pericyte lineage has also been suggested to involve VEGFR2 125, 126, 133, 134. GSCs exposed to hypoxia and/or endothelial cell media expressed vascular markers including CD31 and formed tubes similar to endothelial cells *in vitro*. Experiments with fluorescently labeled GSCs demonstrated that GSCs can incorporate into the vasculature of GBM xenografts. However, the lack of endothelial cells with common driver mutations present in GSCs suggests that transdifferentiation is very rare in human GBMs 125, 126, 134.

VEGF production may also be regulated by the pro-angiogenic chemokine CXCL12, also known as stromal cell-derived factor 1, in GSCs. CXCL12 is highly expressed in GSCs 122 and stimulates GSC VEGF production via the PI3K/AKT signaling pathway 135. The CXCL12 receptors, CXCR4 and CXCR7, are also overexpressed within GSCs 135, 136, and the CXCR4 inhibitor AMD3100 reduced GSC-mediated tumor growth and angiogenesis in association with lower VEGF production 135. GSCs expressing CXCR4 have been identified in close proximity to tumor vascular capillaries, strengthening the idea that these cells are involved in vascular remodeling in the tumor. Recent evidence also strongly implicates the CXCL12/CXCR4 pathway in the growth of GBM cells and GSCs under hypoxia 137 through maintaining GSC self-renewal. CD133-positive GSCs expressed higher levels of CXCR4 mRNA and protein compared to CD133-negative cells, indicating that chemokines target GSCs inducing a migratory response. These data highlight evidence that GSC mediated angiogenesis is regulated by VEGF and CXCL12 signaling.

## Hypoxia and GSC invasion

Solid tumor cells in a hypoxic niche contribute to tumor aggressiveness and metastases, with specific roles for cancer stem cells that can have epithelial-to-mesenchymal transition (EMT) phenotypes 121, 138. While cancer stem cell-mediated invasion and metastasis are more commonly studied in epithelial tumors, GSCs have been shown to be more migratory and invasive than their non-GSC counterparts 139. Understanding the mechanisms responsible for this effect is important as the invasive nature of GBMs makes them very difficult to completely resect.

Hypoxia is an important modulator of GSCs in the context of epithelial to mesenchymal transition, which often leads to greater migration and invasion and ultimately, tumor recurrence 121. When non-mesenchymal GBM lines were exposed to hypoxia, a mesenchymal shift resulted in greater invasive capacity. The morphological change was inhibited by knockdown of HIF1α and the EMT transcription factor ZEB1. Further evidence of a hypoxia-induced mesenchymal shift in GBM was seen through the co-localization of GLUT1, ZEB1, and the mesenchymal marker YKL40 in hypoxic areas 140. Furthermore, hypoxia-activated A_3_ Adenosine Receptor was demonstrated to promote the migration/invasion of GSCs via a HIF-2-dependent mechanism 141.

The increased invasiveness of GBMs in hypoxic condition has also been linked to their enhanced hyaluronic acid production 142. Hyaluronic acid (HA) is one of the main extracellular matrix (ECM) components in the brain, and higher levels of HA have been linked to the invasive edge of GBMs 143. GSCs have been identified by their distinct expression of stem-marker and HA receptor, CD44, which, in addition to maintenance of stem-like properties 144, contributes to the migratory and invasive capability of GSCs 145. The integration of these observations has led to experimental targeting of HA in GSCs to investigate the effect on therapy-resistant GSCs 146.

A recent study focused on the importance of recombination signal binding protein for immunoglobulin kappa J (RBPJ, CBF1). CBF1 is a master transcriptional regulator of the notch signaling pathway and contributor of GSC maintenance as well as an important regulator of EMT in GSCs 147. In patient tissue, CBF1 is a clinically predictive biomarker, but its expression is heterogeneous within the tumor tissue and likely marks those cells that have undergone EMT and likely to be more invasive and resistant to chemotherapeutics. Structural changes within cells at the invasive edge are influenced by hypoxia-induced Cyclin G2 that facilitates membrane ruffles for directing cellular movement. Cyclin G2 is seen *in vivo* in abundance in areas of pseudopalisades, where glioma cells are actively migrating 148. Although additional research is needed, hypoxia-induced changes in migration may also be a consequence of alterations to mitochondrial dynamics. More recent evidence specifically implicates Drp1 in GSC maintenance, with higher levels of an activating phosphorylation in the GSC fraction 149. Together, these data suggest important links between GSC invasion and hypoxia, mesenchymal phenotypes, and the extracellular matrix.

## Hypoxia and acidic stress regulation of the GBM epigenome

Chromatin remodeling regulates the GSC state as well as therapeutic resistance, and a rapidly developing area of interest is regulation of the epigenetic landscape by the hypoxic tumor microenvironment. While it is known that there are many epigenetic alterations that are involved in the initiation and progression of brain tumors, much less is known about how hypoxia contributes to these mechanisms. The discovery that expression of mixed-lineage leukemia 1 (MLL1), a histone methyltransferase specific for the lysine 4 methylation of histone H3, is increased in GSCs by hypoxic conditions has driven interest in this field. MLL1 enhances hypoxia response gene expression, including VEGF, by enforcing expression of HIFs, mainly HIF2α, in hypoxia 150. Recently, it was also shown that the induction of the Ten-eleven Translocation (TET) family of DNA demethylases by hypoxia promotes the expression of the stem cell genes OCT4 and NANOG in glioma cells. TET1 and TET3 were shown to bind specifically to the genomic regulatory regions of these genes and actively demethylate these regions, which led to increased expression of these pluripotency genes and ultimately increased formation of GSCs 151.

In GSCs, there are also established roles for some Jumonji Domain-Containing proteins that modify histones and are known to be oxygen dependent. GSCs that survived kinase inhibitor treatment had differential H3K27me3 profiles, and the Jumonji Domain-Containing Protein 3 (JMJD3)/Lysine Demethylase 6B (KDM6B) was implicated in cellular maintenance 152. JMJD3/KDM6B was also important for maintaining GSC neurosphere formation potential due in part to regulation of STAT3 activity 153. GSK-J4, a JMJD3 inhibitor, was recently shown to inhibit glioma cell growth in association with elevation of H3K27me3 154, and targeting KDM4A reduced glioma cell survival via increased autophagy 155. Thus, there are multiple lines of evidence that hypoxia regulates DNA and histone methylation that is important for GSC maintenance.

Hypoxia and acidic stress also repress the epigenetic modifier chromodomain-helicase-DNA-binding protein 7 (CHD7) 101. CHD7 is one of a family of CHD proteins involved in transcription, chromosomal stability, and DNA repair 156, 157, which binds methylated histone H3 lysine 4 (H3K4me) 158. In mice, CHD7 appears to have the capability of both enhancing and inhibiting embryonic stem cell genes by co-localizing with OCT4, SOX2, and NANOG on enhancer regions 159. CHD7 is mutated in a congenital disorder called CHARGE syndrome 160, 161, but very little is known about CHD7 and cancer 162-164. However, studies showed CHD7 binding within 10kb of VEGF as well as increases in VEGF transcription upon loss of CHD7 in mice 159, 165, suggesting a potential role in angiogenesis. We recently reported that CHD7 was repressed by acidic stress and that CHD7 targeting in GBM cells promotes angiogenesis as determined by increased tube formation 101. These data suggest that additional investigation of hypoxia and acidic stress effects on epigenetic modifiers may identify further mechanisms through which these tumor microenvironments may impact cell state.

## Interactions of the immune system and GSCs in the hypoxic niche

Although we have thus far focused on direct effects of hypoxia on GBM cells to regulate tumor growth, the tumor microenvironment also impacts GBM cells indirectly via paracrine effects mediated through nearby non-neoplastic cells. Under a hypoxic microenvironment, macrophages undergo phenotypic changes that activate the expression of mitogenic and proangiogenic cytokines and enzymes 166. This enhances tumor progression, angiogenesis, and metastasis 166. The immunosuppressive activity of tumor-associated macrophages (TAMs) is enhanced in solid tumors when HIF1α is upregulated 167. GSCs promote TAM immunosuppressive phenotypes via mechanisms involving cytokines such macrophage inhibitory cytokine-1 (MIC-1) and Transforming Growth Factor β (TGF-β) and the transcription factor STAT3 168. GSCs also efficiently recruit TAMs including through secreting periostin 169, a protein that serves as an integrin ligand 29. Periostin secretion levels positively correlated with TAM numbers and silencing periostin in GSCs resulted in decreased TAM density, decreased tumor growth, and increased survival of mice bearing GSC-derived xenografts 29. While this model suggested periostin-mediated GSC and TAM co-localization in the perivascular niche, hypoxia is known to increase periostin in glioma cells to promote macrophage recruitment through mechanisms involving TGF-β 170.

Macrophage migration inhibitory factor (MIF) is a type of cytokine released by leukocytes. MIF levels have been associated with immunosuppression as well as angiogenesis, cell differentiation, and cell proliferation in tumor cell lines 171. Immunohistological analysis of MIF in GBM tissues demonstrated a large accumulation of MIF protein in necrotic areas and tumor cells surrounding blood vessels 171. Under hypoxic stress, the MIF gene was transcriptionally upregulated, leading to elevated MIF mRNA as determined in Northern analysis 171. While experiments have largely focused on a GBM-cell-intrinsic role for MIF signaling, additional data demonstrated MIF promoted mast cell migration to GBMs 172. Recent evidence also indicated that GSC-derived MIF increased the activity of myeloid derived suppressor cells (MDSCs) 173 to suppress the immune system, and it has been shown that these cells accumulate in GBM patients 174. In contrast, the MIF receptor CD74 was shown to be restricted to TAMs where it appeared to promote a proinflammatory phenotype and was associated with improved patient outcomes 175. LGALS1 (galectin-1) and IGFBP2, which are upregulated in GBM and correlate with poor patient outcomes, have been identified with a subset of genes involved with immunosuppression. They have also been shown to be positive regulators of MDSC and immunosuppressive macrophages, which could provide an explanation for MDSC accumulation in GBM 176, 177. Interestingly, IGFBP2 increases neural stem cell and GSC maintenance 178, 179, providing additional links between stem cell and immune phenotypes.

Gliomas have been characterized as immunologically “cold” tumors that have a highly immunosuppressive microenvironment, particularly in terms of adaptive immunity. Using immunogenomic characterization data compiled by The Cancer Genome Atlas (TCGA), Thorsson et al. characterized these tumors as immunologically quiet and lymphocyte depleted, as the current understanding of immune activity in these tumors is that it is largely driven by monocytes and innate immunity 180. HIFs play a role in the immunosuppressive environment as it has been shown that HIF-1α encourages the migration of T regulatory cells (Tregs) in the presence of hypoxia, and HIF-1α knockout in Tregs enhanced survival in a murine model of glioma, indicating that this response is important for immunosuppression and tumor progression 181. Considering the increasing importance of immunotherapy-based approaches, these data highlight the critical need to better model the effects of hypoxia on tumor-associated immune populations.

### Hypoxia promotes GSC therapeutic resistance

The hypoxic microenvironment of GBMs has many effects that result in tumor cell resistance to chemotherapy and radiation. These include effects on DNA repair, DNA stability, ABC transporter expression, cell cycle checkpoint protein expression, and vasculature function. As a broad indicator of GSC survival in the hypoxic microenvironment, the CD133-positive GSC fraction are more resistant to apoptosis under hypoxia 182. GSCs are also less sensitive to irradiation and chemotherapy induced cell death, suggesting that tumor recurrence is mediated by GSCs 10, due in part to hypoxia-mediated GSC maintenance 183.

Changes in the ability to repair DNA in the hypoxia microenvironment lead to therapeutic resistance 184. Short-term hypoxic conditions activate DNA damage signaling pathways, with therapeutic resistance possibly due to increased activation of checkpoint proteins 185. Long term effects of hypoxia lead to downregulation of DNA repair pathways that may promote genetic instability: these include DNA double-strand break repair, mismatch repair, and nucleotide excision repair 186. The lack of oxidation of DNA free radicals that occurs when oxygen tensions are low also prevents the damage and breakage of DNA 187, 188. While these pathways have not all been investigated in the context of hypoxia in GBMs, GSCs are known to have increased activating phosphorylation of ATM and checkpoint proteins as well as increased levels of some DNA repair proteins, which enables the cells to more rapidly repair damaged DNA 10, 189. However, the fidelity of this repair is unclear: once arrested cells continue though the cell cycle, secondary tumors may arise from the damaged cells 185. Furthermore, elevation of proliferating cell nuclear antigen associated factor (PAF) in GSCs may facilitate DNA damage tolerance via translesion DNA synthesis 190. Quiescent populations of GSCs that are not replicating are also relatively insensitive to DNA damaging agents 191. The suppression of DNA repair under a hypoxic microenvironment could be a potential cause for the genetic instability of cancer cells that drives the progression of brain tumors 184. These results support the hypothesis that hypoxia induces drug resistance by preventing drugs from damaging the DNA of tumor cells 188.

Cell surface transporter proteins are considered important mechanisms of drug resistance and are affected by hypoxia in GSCs. The ATP-binding cassette (ABC) transporters are a class of proteins that have a wide range of biological effects. These transporters promote therapeutic resistance by removing chemotherapy from the tumor cells, minimizing the time that the drugs have to be effective 192. The stem cell transcription factor OCT4, which is known to be HIF2α-regulated, increases expression of ABCG2 in GBM cells 193. The cyclic hypoxic microenvironment (as opposed to chronic hypoxia) in GBM cells has also been shown to increase ABCB1 expression via HIF1α promoter binding, enhancing its expression 192. The greater expression of the ABC transporters, in turn, strengthens chemoresistance 192. Furthermore, ABCB5, which has been suggested to be a CSC marker in some tumors, is expressed in GSCs and mediated TMZ-resistance in GBM cells 194.

Hypoxic regions form when tumor cells lack proximity to a functional blood vessel leading to low oxygen tensions. Cells located at least 70 µm away from the nearest functional blood vessel do not receive adequate amounts of oxygen, leading to a conversion to a hypoxic cell state 195. Because chemotherapies reach tumor cells via the circulatory system, the distance cells are to the nearest blood vessel has an important effect on the efficacy of the drug 196, 197. This, combined with the need for drugs in the brain to cross the blood brain barrier, often results in drug penetration that is lowest in brain tumors especially compared to cancers of the heart, kidney, and liver 198. While the blood brain barrier is disrupted in GBM, the blood brain barrier can remain intact in brain adjacent to tumor where the cells responsible for recurrences have dispersed, preventing chemotherapies from reaching critical tumor cells.

### Diagnostic and therapeutic modalities targeting acidic stress and hypoxia

**Figure 4:**
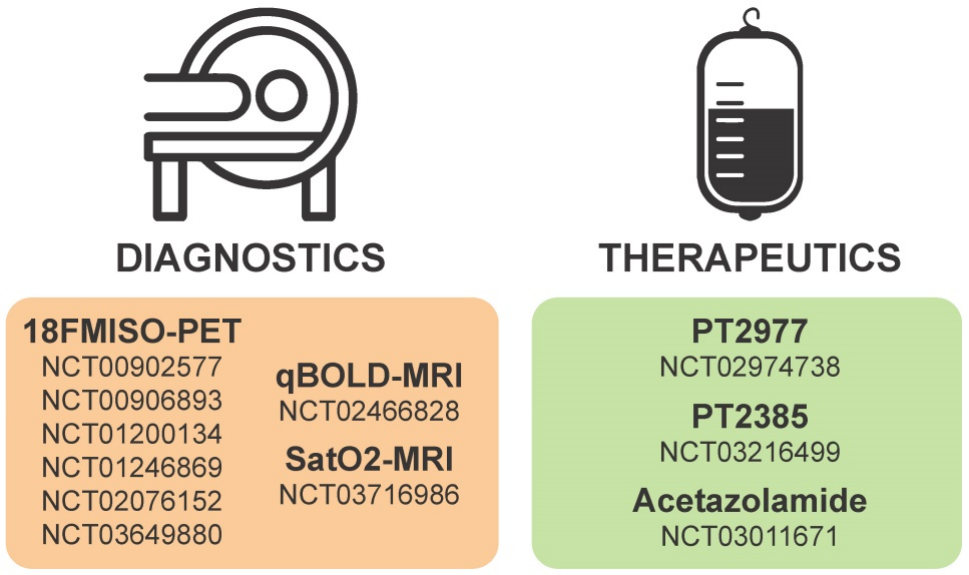
** Hypoxia or HIF-based diagnostics and therapeutics currently in clinical trial for gliomas.** Numbers in each section correspond to studies listed as current on clinicaltrials.gov.

Diagnostic monitoring of tumor progression post-resection can be complicated by ischemia, and there are a number of clinical trials (see **Table 1** and** Figure 4**) focused on improved imaging modalities that identify tumors by regions of hypoxia or acidosis. An innovative study synergized radiomic features in MRI scans and RNA expression data from hypoxia markers to generate a hypoxia enrichment score (HES) that was highly predictive of the survival of GBM patients 199. Therapeutic interventions targeting acidic stress are rare, and diagnostic procedures to measure the acidic microenvironments *in vivo* have proven challenging to develop but have recently gained traction. Cutting-edge MRI techniques that highlight regions of acidosis are being developed as novel imaging tools to improve patient care and explore the pathophysiology of brain tumors 200-202. Using this technique, rat brain tumors treated with TMZ, the first-line chemotherapy approved for standard of care in human GBM patients, were shown to have a normalized intratumoral pH, indicating the ability of TMZ to modulate pH in solid brain tumors 203. However, there has been little follow-up on the concept of extracellular pH modulation by TMZ in GBM. In the authors' opinion, acidic stress is an important contributor to the GBM tumor microenvironment and should be considered as a therapeutic target. Diagnostic procedures that are developed to incorporate estimation of tumor hypoxia and/or acidity could prove useful in stratification of patients for personalized therapies in the future.

Considering the critical roles of HIF1α and HIF2α in cancer biology, many strategies have been considered to target HIFα signaling in solid tumors including GBMs. Targeting hypoxia/HIFα signaling is thought to be a viable strategy to sensitize GSCs to radiation and chemotherapy as well as to inhibit the pro-tumorigenic biology induced when blood vessel collapse occurs with anti-angiogenics 44, 204. Inhibition could be mediated by therapies that promote oxygenation, decrease HIFα stability, prevent HIFα DNA binding, or inhibit the downstream mediators of pro-tumorigenic hypoxia/HIF effects. A current list of ongoing clinical trials targeting hypoxia in brain tumors is illustrated in **Figure 4** and additional details are provided in** Table 1**.

HIF-1α has been shown to reach maximal expression levels in the brain after 5 hours of hypoxia exposure, but returns to basal levels at 12 hours 205. The activators responsible for HIF1 transcription are CREB binding protein and p300, both of which interact with the carboxy-terminal transactivation domain of HIF1 206. As a result, HIF1α activation promotes the expression of numerous gene products. These include pluripotency-associated transcription factors like OCT3/4, NANOG, and SOX2; glycolysis- and EMT-associated molecules like CXCR4, SNAIL, and TWIST; microRNAs; and angiogenic factors such as VEGF 207. These gene products lead to increased self-renewal ability, tumor survival, distorted energy metabolism, invasion, angiogenesis, and treatment resistance 207. Because HIF1α activates these gene products, targeting HIF1 is a potential therapeutic strategy to target GSCs 207. However, it is important to consider the potential for side effects against neoplastic neural stem cells when targeting GSCs, as hypoxia is present in neural stem cell niches and HIF1α regulates neural stem cell proliferation and differentiation 208.

Targeting of HIF expression or activity could occur through multiple mechanisms. In GBM, the cardiac glycoside digoxin has been shown to inhibit HIF1α expression in hypoxia 209. Digoxin treatment reduced expression of CD133 and decreased neurosphere formation, suggesting this clinically utilized drug may be able to target GSCs. Importantly, a clinical trial (clinicaltrials.gov identifier NCT03216499) is currently recruiting recurrent GBM patients for treatment with the HIF2α specific inhibitor PT2385, which was effective against renal cell carcinomas in preclinical studies 210, 211. Of note, recent work has also shown that the FDA approved drug topotecan may in fact be capable of targeting HIF1α via perturbations in levels of SUMOylation thereby altering the stability/degradation of HIF 212.

The canonical hypoxia-induced gene is the proangiogenic factor VEGF that is elevated in GSCs 11. Therapies have been developed to target VEGF activity by binding the ligand (the VEGF antibody bevacizumab) and inhibiting the receptor with varying degrees of specificity (sorafenib, sunitinib, etc.) to normalize the tumor vasculature 213. Neutralizing anti-VEGF antibody was previously shown to extend the survival of GBM bearing mice 11, 214. While these initial studies suggested that treated tumors had reduced vasculature and increased apoptosis 214, subsequent results demonstrated increased tumor cell invasion 215. Furthermore, treatment of GBM patients with bevacizumab did not improve patient survival 216. Another potent angiogenic chemokine, SDF-1a, and its cognate receptor, CXCR4, are highly expressed in hypoxic regions and have been used to develop hypoxia-targeted drug delivery mechanisms. Nanoparticle induced CXCR4-overexpressing human adipose cells have been tested in GSC organoid models and murine models: they effectively home to the necrotic core of tumors and could serve as an effective delivery mechanism for nanoparticle-based drug treatments 217. CXCR4 inhibition in combination with anti-VEGF therapies has been tested in animal models with some success, and is another possible therapeutic modality to target angiogenic responses within the tumor microenvironment 218. Another innovative study combined engineered liposomal delivery that targets brain microvascular endothelial cells. Using the low-density lipoprotein receptor-related protein-1 and a hypoxic prodrug radiosensitizer in animal models, radiosensitivity was increased by enhancing the DNA damaged caused by ionizing radiation 219.

Mammalian target of rapamycin (mTOR) is an intracellular kinase that regulates cell growth and cell cycle progression via signaling from nutrients and growth factors 220. Previous studies have shown that mTOR is deregulated in GBM contributing to radiosensitization. To combat this problem, Kahn et al. demonstrated that exposing the tumor cells to AZD2014, an mTORC1 inhibitor, increased the radiosensitivity of GSCs. Additionally, clonogenic survival analysis showed that CD133-positive and CD15-positive GSC cells exposed to this mTORC1 inhibitor at least one hour before irradiation increased sensitivity to radiation 221. These data suggest that mTORC1 inhibition could target GSCs, which serve as a reservoir for radioresistance.

Mitogen-activated protein kinase (MAPK) can be activated by stress signaling such as that resulting from hypoxia 222. In general, the RAS-MAPK pathway plays a role in cell development, cell cycle regulation, tumor formation, and metastasis 223. When activated, RAS then activates RAF kinase, which then activates downstream MAPK signaling 224. The altered activity of the RAS/MAPK signaling pathway leads to abnormal cell growth and proliferation, as well as initiating other abnormal cellular behaviors like invasion and apoptosis 224. Expression of constitutively active Ras in the mouse subventricular zone led to the development of gliomas through Ets transcription factor-dependent mechanisms 225 (and Ets binding elements are in HIF2a target genes). However, rather than RAS mutations, activation of this pathway in GBM is frequently due to amplification or constitutive activation of receptor tyrosine kinases including Epidermal Growth Factor Receptor (EGFR) and Platelet Derived Growth Factor Receptor (PDGFR): further elevation of ligands and/or receptors under hypoxia influences even greater activation of the RAS/MAPK signaling pathway 224, 226, 227. Thus, targeting the RAS protein with chemotherapy in GBM may be useful because of its high expression and its association with tumorigenesis 224. Further downstream of MAPK, ERK signaling can regulate AMP-activated Protein Kinase (AMPK), which is an important regulator of cellular bioenergetics via activation of catabolism to generate ATP 228. The AMPK stress-induced pathway is hijacked by GSCs for their adaptation to tumor-related stressors in the microenvironment through the Cyclic AMP-Responsive Element-Binding Protein 1 (CREB1) transcriptional program that controls both HIF-1 and GA Binding Protein Transcription Factor Subunit Alpha (GABPA) expression. This pathway has been successfully targeted in mouse models with AMPK tissue-specific and whole-animal knockouts, which prompts interest in developing specific AMPK inhibitors for glioma therapy 229.

Nitro compounds, N-oxides, and quinones are bioreductive prodrugs that target the hypoxic tumor cells by being reduced by intracellular oxidoreductases in an oxygen-sensitive manner to form cytotoxins 230. While hypoxia causes tumors to become resistant to radiation, bioreductive drugs are used as antimicrobials, chemotherapeutic agents, and radiation sensitizers 231. AQ4N is an N-oxide that has been shown to have an anti-tumor effect on hypoxic tumor cells 232 and has been used in phase I clinical trial in GBM patients 233. AQ4N metabolizes to AQ4 that binds non-covalently to DNA to initiate anti-tumor effects 230, 232. AQ4 can then inhibit topoisomerase activity as tumor cells begin to re-enter the cell cycle 232. Tirapazamine (TPZ) is another N oxide which was shown to selectively kill cells in hypoxic environments 234. TPZ has been used in a phase II clinical trial in GBM patients, unfortunately providing no significant survival advantage 235. As TPZ has poor extravascular penetration 236, more optimized analogues including SN30000 have been developed. While SN30000 has not been extensively studied in GBM, the compound can cross the blood-brain-barrier 237, suggesting possible efficacy in patients.

The quinone mitomycin C (MMC) is activated under hypoxic conditions in tumor cells 238. In glioma, MMC combined with recombinant adeno-associated virus II resulted in reduced tumor growth *in vivo* 239. MMC-mediated GBM cell death was also increased when cells were pretreated with the DT-diaphorase inducer, dimethyl fumarate 240. As a reductase, DT-diaphorase is known to activate quinones like MMC and is elevated in GBMs 241. Thus, MMC treatment may take advantage of the elevated DT-diaphorase levels in GBM to provide a greater therapeutic window and molecular analysis of DT-diaphorase levels may provide a biomarker for MMC therapeutic response.

## Concluding Statement

Glioblastoma remains a formidable tumor that is notoriously difficult to prevent from recurrence, which contributes to abysmal survival in patients. Compared to solid tumors outside the brain, glioblastoma treatment is also more complex due to the presence of the blood-brain-barrier: development of certain types of treatment modalities is precluded without novel delivery methods that are able to circumvent the restrictions of the blood-brain-barrier. For example, many conventional chemotherapies and antibodies that target antigens in and on the surface of tumor cells have difficulty crossing an intact blood-brain-barrier, although there are antibodies that work well in the bloodstream without having to cross into the tumor (bevacizumab, nivolumab, pembrolizumab and ipilumimab for example). While the tumor associated blood-brain-barrier is remarkably fenestrated and is constantly being remodeled, the blood-brain-barrier in regions where invading tumor cells reside may be intact preventing eradication of the disease. Continual failures with novel treatment strategies suggest that there are major characteristics of these tumors that are not adequately being modeled for drug testing *in vitro* or that we still do not fully understand. It is of our opinion that more accurately modeling the tumor microenvironment *in vitro* and consideration of its links to differentiation state and therapeutic resistance will allow for more efficient transfer of novel therapeutics from *in vitro* studies to the clinic. In addition, added emphasis on targeting these microenvironments during therapeutic design may lead to desirable improvements in standard of care. Considering this, hypoxia and acidic stress are major micro-environmental stresses that occur commonly in GBM solid tumors. This is especially important when considering the GSC niche that can be found within hypoxic and acidic zones. Establishing efficient models of these microenvironments *in vitro* and targeting these *in vivo* to exploit resistant cell populations, i.e. GSCs, is of substantial importance when identifying novel treatment strategies to synergize with standard of care.

## Figures and Tables

**Table 1:** Details of hypoxia or HIF-based diagnostics and therapeutics currently on clinicaltrials.gov.

Intervention	Mechanism	Trial ID	Title	Phase	Start year	Status
^18^F-FMISO PET 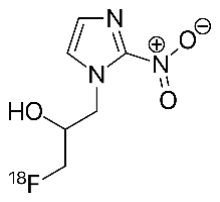	PET radio-tracer for imaging hypoxia	NCT00902577	Multicenter, Phase II Assessment of Tumor Hypoxia in Glioblastoma Using 18F-Fluoromisonidazole (FMISO) With PET and MRI	Phase 2	2009	Completed
		NCT00906893	Methodological Evaluation of Fluor 18 Labelled Fluoromisonidazole ([18F]-FMISO) Positon Emission Tomography-Computed Tomography (PET-CT) for Non Operated Glioblastoma	Phase 2	2009	Completed
		NCT01200134	Hypoxia Diagnosis and Evaluation Using F-MISO PET and Biomarkers in Brain Tumors	Phase 2	2010	Completed
		NCT01246869	Assessment of Primary and Metastatic Brain Tumor Hypoxia With 18F-Fluoromisonidazole, [18F]Fluoro-2-deoxy-D-glucose (FDG) and [15O]Water (H215O)	N/A	2010	Recruiting
		NCT02076152	A Study to Evaluate Vascular Normalization in Patients With Recurrent Glioblastoma Treated With Bevacizumab Using FMISO PET and Vascular MRI	N/A	2014	Completed
		NCT03649880	Feasibility of FMISO in Brain Tumors	Phase 1I	2018	Recruiting
Ferumoxytol-based qBOLD MRI 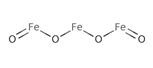	Iron-based Quantitative BOLD MRI for hypoxia detection	NCT02466828	Quantitative Blood Oxygenation Level Dependent (qBOLD) MR Imaging of Glioblastoma Multiforme for Assessment of Tumor Hypoxia	Early Phase 1	2015	Completed
SatO2-MRI	Mapping tissue oxygenation with MRI	NCT03716986	Multimodal Imaging of Hypoxia in Gliomas		2018	Not yet recruiting
PT2977 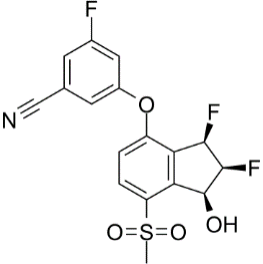	Selective HIF-2α inhibitor	NCT02974738	A Phase 1, Multiple-Dose, Dose-Escalation and Expansion Trial of PT2977, a HIF-2α Inhibitor, in Patients With Advanced Solid Tumors	Phase 1	2016	Recruiting
PT2385 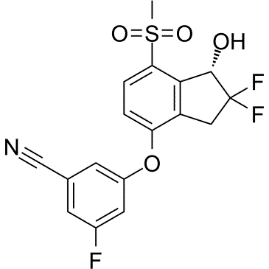	Selective HIF-2α inhibitor	NCT03216499	Single-Arm, Open-Label Phase II Efficacy Study of First-in-Class HIF2-Alpha Inhibitor, PT2385, for Patients With Recurrent Glioblastoma	Phase 2	2017	Active, not recruiting
Acetazolamide 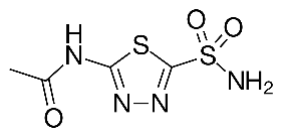	Carbonic anhydrase inhibitor	NCT03011671	A Phase I Study of Safety and Tolerability of Acetazolamide With Temozolomide in Adults With Newly Diagnosed MGMT Promoter-Methylated Malignant Glioma	Phase 1	2018	Recruiting

## Abbreviations

GSC: glioma stem cell
GBM: glioblastoma
TMZ: temozolomide
FACs: fluorescence activated cell sorting
VEGF: vascular endothelial growth factor
AKT: protein kinase B
HIF: hypoxia inducible factor
ARNT: aryl hydrocarbon receptor nuclear translocator
VHL: von Hippel Lindau factor
HRE: hypoxia response elements
MAX: Myc associated protein
EPO: erythropoietin
EDN1: endothelin 1
GLUT1: glucose transporter 1
MYC: MYC proto-oncogene
HGF: hepatocyte growth factor
CAIX: carbonic anhydrase IX
PHD: prolyl hydroxylase domain protein
WAF1: cyclin-dependent kinase inhibitor p21
EMT: epithelial to mesenchymal transition
RBPJ, CBF1: recombination signal binding protein for immunoglobulin kappa J region
DRP1: dynamin related protein 1
MLL1: mixed lineage leukemia 1
TET: ten-eleven translocation methylcytosine dioxygenase
MAPK: mitogen-activated protein kinase
MTOR: mammalian target of rapamycin
CREB1: CAMP responsive element binding protein 1
TPZ: tirapazamine
MMC: mitomycin C
EGF: epidermal growth factor
PDGF: platelet derived growth factor
ECM: extracellular matrix
AMPK: AMP-activated protein kinase
HA: hyaluronic acid
